# Analysis of the genome-editing activity of microinjected CRISPR/Cas9 ribonucleoprotein complexes in *Daphnia pulex*

**DOI:** 10.17912/micropub.biology.001310

**Published:** 2024-10-30

**Authors:** Megan E. Maar, Jay C. Miller, Michael Lynch, Andrew C. Zelhof

**Affiliations:** 1 Biology, Indiana University, Bloomington, Indiana, United States; 2 Center for Mechanisms of Evolution, Arizona State University, Tempe, Arizona, United States

## Abstract

Although *Daphnia* is a widely used model organism with a completely sequenced genome, molecular tools for analyzing specific gene functions are still being developed. Progress has been made in developing CRISPR/Cas9 gene editing in *Daphnia* . However, the gene-editing activity of injected ribonucleoprotein complexes (RNPs), the success of co-injected RNPs with different gRNAs, and the heritability of mutations in asexual progeny need further investigation. Here, we show prolonged Cas9 RNP activity past the one-cell stage injected individuals, leading to a wide range of somatic mutations, and germline mosaicism of heritable biallelic mutations.

**Figure 1. CRISPR/Cas9 editing of the scarlet locus.**
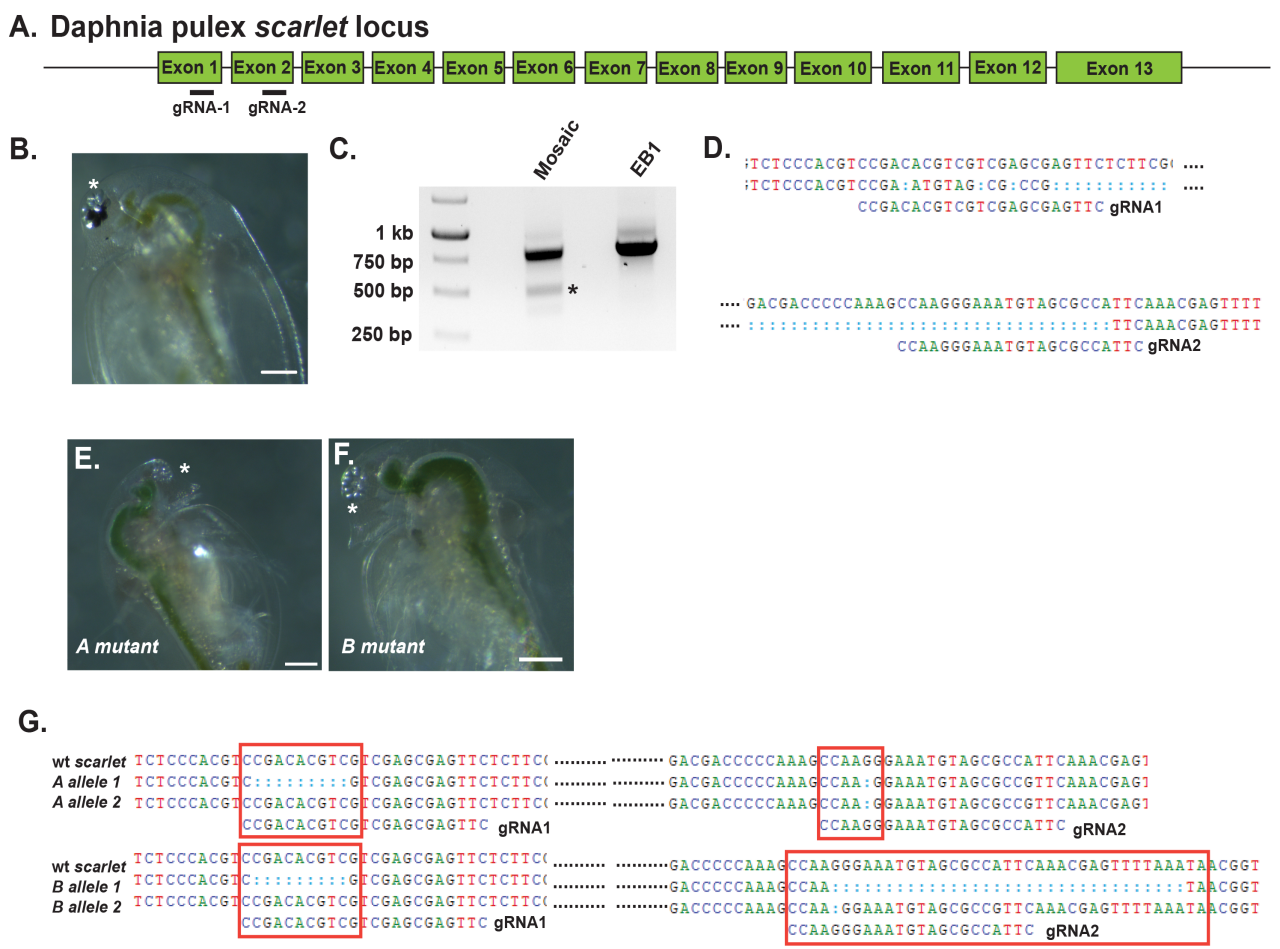
**A.** Schematic of the *Daphnia pulex* *scarlet* locus with gRNA1 located in exon 1 and gRNA2 in exon 2. **B.** Injected G0 *scarlet* mosaic *Daphnia* . The asterisk marks the partial lack of pigment in the ommatidium of the eye. **C.** PCR amplicons of the *scarlet* genomic region flanking both gRNAs from (left) the injected *scarlet* mosaic versus (right) a wild-type EB1 pigmented individual. Both individuals contain the expected band size of 789 bp. Small deletions and insertions are not detected visually on a 1% agarose gel. The mosaic individual had a second prominent band (asterisk) the size equivalent to a deletion spanning the two gRNAs, 512 bp. **D.** Sequence confirmation that the lower PCR amplicon represents a deletion between the two gRNA sites. Blue dots represent deleted sequences, and black dots represent sequences not changed. **E-F.** The mosaic-injected individual produced two *scarlet* mutants, A and B. Both mutants have a total lack of pigment in the ommatidia. **G.** Sequence confirmation of induced changes at the gRNAs in the A and B *scarlet* mutants compared to wild-type. The red boxes highlight the change at each gRNA, the blue dots represent deleted sequences, and the black dots represent sequences not changed. Scale Bars 200um.

## Description

The aquatic microcrustacean *Daphnia pulex* , commonly known as the water flea, has been a widely studied model organism in ecology (Ebert, 2022) , ecotoxicology (Iguchi et al., 2006) , evolutionary genetics (Pfrender & Lynch, 2000) , and developmental genomics (Mahato et al., 2014) . In 2011, *Daphnia* was the first crustacean species to have its entire genome sequenced (Colbourne et al., 2011) , and in 2016, a more high-quality reference genome assembly was established and additonal genomes added in subsequent years (Lynch et al., 2024; Ye et al., 2023; Ye et al., 2017) . Furthermore, there have been advancements in targeted genetic modification in *Daphnia* through gene silencing using RNA interference (Hiruta et al., 2013) , gene editing using TALEN (Hiruta et al., 2014) , and CRISPR/Cas9 (Hiruta et al., 2018) , helping establish the molecular tools to characterize specific gene functions. Because *Daphnia* reproduce by parthenogenesis under most conditions, microinjection into the asexual embryos is an efficient delivery method for gene editing and allows for the easy establishment of clonal mutant lines. Recent advances in the microinjection technique and genetic analysis of CRISPR/Cas-induced mutations and heritability (Xu et al., 2024) have helped establish CRISPR/Cas gene editing in *Daphnia* . Here, we present findings that further elucidate the genetic modification activity of injected CRISPR/Cas9 ribonucleoprotein complexes (RNPs) in the G0-injected *Daphnia* and the biallelic genetic heterogeneity in the parthenogenically produced progeny.

Cas9 RNPs are directed by single guide RNAs (sgRNAs) to the complementary genomic sequence of the sgRNA next to the protospacer adjacent motif (PAM), where the Cas9 nuclease cleaves and induces a double-stranded break (DSB). DSBs can be repaired by the endogenous nonhomologous end-joining pathway (NHEJ), leading to deletion or insertion mutations, inducing reading frameshifts, and gene deactivation. Another repair pathway that can fix DSBs is homology-directed repair (HDR). While HDR typically uses the homologous sister chromatid as a template to precisely repair the DSB, exogenous DNA sequence containing homology that surrounds the RNP target site can compete as the repair template. This process has been invaluable for precise genome editing and introducing large exogenous DNA sequences of interest in *Drosophila* and mouse ES cells. Moreover, previous work has shown that the HDR pathway is more efficient when two RNPs with separate gRNAs are utilized (Acosta et al., 2018; Gratz et al., 2014) .

To test the efficacy and activity of co-injecting two Cas9 RNPs in *D. pulex* , we injected two RNPs with sgRNAs designed for exons 1 and 2 of the *scarlet* locus ( Figure 1A ). The *scarlet* gene encodes an ATP-binding cassette (ABC) transporter protein necessary to transport tryptophan into the ommatidia of the eye, a necessary precursor for brown and black pigment production (Ewart & Howells, 1998) . Successful biallelic induction of DSBs at the *scarlet* locus and gene deactivation lead to the loss of pigment in the ommatidia and ocellus (Ismail et al., 2018) and a clear phenotypic marker of successful Cas9-induced mutations.

Embryos were injected within 15 minutes after ovulation to deliver the RNPs into the embryo while still in the single-cell stage (Hiruta et al., 2010) and also to ensure that the embryonic membrane was elastic enough to be repaired after injection (Kato et al., 2011) . The injected embryos were monitored for loss of pigment in the developing eye. Of the 13 injected individuals that survived and developed into neonates, we detected one *scarlet* mosaic with both pigmented and non-pigmented ommatidia ( Figure 1B ). This indicates that the Cas9 RNPs remained active past the single-cell stage. The *scarlet* mosaic survived to produce only one clutch of four progeny, a marker of suboptimal health. This could be from the direct effects of injection, Cas9 RNP activity and potential off-target mutations, or other environmental growing conditions. Of the four progeny, two had a complete loss of pigment in the ommatidia and ocellus, labeled A and B (Figures 1E and 1F), and two had full pigmentation, designating germline mosaicism in the injected individual. PCR of the region flanking both gRNAs in the injected individual revealed the presence of two prominent band sizes ( Figure 1C ). Subcloning of the amplicons and sequencing shows that the upper band (~750 bp) represents several indel mutations at both gRNAs, including both small deletions and insertions, and the lower band (~ 500 bp) represents a complete deletion of the region between both gRNAs ( Figure 1D ). These results show the prolonged and effective activity of each Cas9 RNP at both gRNA targets.

We isolated the A and B *scarlet* progeny to reproduce and create clonal stocks. Individuals ~4 generations out were used for PCR and subcloning of the *scarlet* region. Surprisingly, the A and B lines have unique biallelic mutations at both gRNAs. The A line contains one allele with a 9-bp deletion at gRNA1 coupled with a single bp deletion at gRNA2 and another allele wild-type at gRNA1 coupled with a single nucleotide deletion at gRNA2. The B line contains one allele with the same 9-bp deletion at gRNA1 coupled with a 33-bp deletion at gRNA2 and a second allele wild-type at gRNA1 coupled with a single bp deletion at gRNA2. Each of the indel mutations causes a frameshift, consistent with the loss of function of *scarlet* and total lack of pigment in the ommatidium and ocellus observed. The difference between the mutations observed in the G0 *scarlet* mosaic and the two *scarlet* knockout progeny highlight the prolonged activity of the two Cas9 RNPs in the injected individual and a variety of induced somatic mutations through independent editing events. This high RNP activity may be responsible for reduced viability.

Our study yielded results similar to those obtained by a more extensive study conducted by Xu et al. (Xu et al. 2024) . Both studies injected two sets of gRNAs targeting *scarlet* and successfully recovered hemizygous biallelic mutations, as well as precise deletions between the gRNAs, leading to the loss of *scarlet* function. While our study used only Cas9, Xu et al. demonstrated that mutations could be generated using both Cas9 and Cas12a nucleases. Additionally, Xu et al. found no evidence of off-target mutations, as shown by whole genome sequencing, and gene editing did not increase base substitution rates. Regarding HDR, neither study tested a two-gRNA approach. However, Xu et al., using one gRNA and an ssDNA repair template, were able to recover insertions in the target region, though these insertions were imprecise and accompanied by complex local genomic changes. Together, our results and those of Xu et al. underscore that each injected Cas9 RNP acts independently, editing the germline beyond the one-cell stage through separate events. This approach shows promise as a method for developing transgenic *Daphnia* through HDR.

## Methods

***Daphnia* strains and maintenance:** We maintained 5-6 ~2.5-week-old EB1 *Daphnia pulex* in 200 mL of COMBO artificial lake water (Kilham et al., 1998) at 21℃ with a 16:8 (light: dark) photoperiod. *Daphnia* were fed daily with *Ankistrodesmus falcatus* algae (#151955, Carolina Biological Supply *)* . *Daphnia* stocks were changed once a week to prevent overcrowding, keep the *Daphnia* in an asexually reproducing state, and prevent ephippia production.

**Microinjection equipment and materials:** We pulled glass needles (Aluminosilicate Glass (#AF100-64-10, Sutter Instrument) for injection using a micropipette puller (Sutter Instrument Co. Model P-87) using the following settings:Ramp Test 701, Heat 706, Pull 150, Velocity 65, and Time 240. The needles were wet beveled using a micropipette beveler (Sutter Instrument BV-10) using a 30-degree angle. The beveled needles were rinsed with distilled water and dried using an air duster to remove any debris near the tip that could clog the needle. Injections were done using a Nikon Eclipse TE300 microscope and a Narishige IM 300 Microinjector.

**Preparation of CRISPR/cas9 and plasmid injection reagents:** sgRNAs targeting exon 1 and exon 2 of the *scarlet* locus were designed using flyCRISPR https://flycrispr.org . The putative sgRNA sequences were confirmed by PCR and sequencing of the *scarlet* region in EB1. The sgRNAs were synthesized (Integrated DNA Technologies). The sgRNAs were prepared at a 10µM stock concentration per standard manufacturer's protocol and stored at -80℃. The guide RNAs were mixed with Cas9 enzyme (cat. No 1081058, IDT) for a final concentration of 1µM RNPs in injection buffer (0.1 mM sodium phosphate (pH 6.8), 5 mM KCl).

**Embryo collection for injection:** The injection protocol was based on (Xu et al., 2024) with the following modifications. *Daphnia* with pronounced dark ovaries were isolated into individual wells on 9-well spot plates (Cat. No 13-748B, Fisher Scientific) in COMBO and kept at room temperature. *Daphnia* were monitored for molting and subsequent ovulation. After the completion of ovulation, the *Daphnia* were left at room temperature for 5 minutes, then transferred to the inside lid of a small petri dish (60x15 mm X Cat No. 430166, Fisher Scientific) with a glued-down glass coverslip (Cat No. 2850-22, Corning) in a few drops of ice-cold 60mM sucrose in COMBO and placed on ice for 8 minutes. The embryos were then dissected out of the brood chamber for immediate injection.

**Microinjection:** The embryos were aligned against the edge of the coverslip for injection. The glass needle was positioned in the center of the embryo, and proper release of the injection solution was verified through perturbations in the cytoplasm. The injected embryos were left at room temperature for the injection day, and an additional 600µl of 60mM sucrose COMBO was added before moving the embryos to a 21℃ incubator overnight. The following day, the injected embryos were screened to select all intact embryos and were isolated into sterile COMBO. The developing embryos were kept in the dish to be monitored until they could swim 3 days post-injection. All developed injected *Daphnia* were transferred to individual vials in approximately 25 ml COMBO. Developing *Daphnia* were monitored for the presence/loss of pigment in the developing eye. For imaging, *Daphnia* were anesthetized by placing in 10% EtOH for 3.5 minutes, then moving to COMBO. If viability was not a concern post-imaging, *Daphnia* were anesthetized for 8-10 minutes in 10% EtOH and then moved to COMBO. Images were taken on a Zeiss Discovery V12 scope and associated camera. Images were imported into Adobe Photoshop for processing.

**Genetic analysis of *scarlet* mutation:** Genomic DNA was isolated according to kit instructions (Cat. No. D4069, Zymo Research). Flanking primers (5’ctactgatggatcacctccactcgtta3’,5’cattaaagccacgagcgaaccgggttg3’) of the gRNA locations were used to amplify the targeted region of the *scarlet* locus. Subsequent PCR products were either directly sequenced or subcloned before sequencing. The resulting sequence was compared to our genomic reference sequence for EB1 utilizing Sequencher 5.4.6.

## Reagents

**Table:** 

Strain	Genotype	Source
EB1	*Daphnia pulex*	Dr. Sen Xu, University of Missouri

**Table:** 

CRISPR Nuclease	Description	Available From
Cas9	Alt-R™ S.p. Cas9 Nuclease V3	IDT

**Table:** 

CRISPR guide RNA	Sequence (5’→3’)	Synthesized From
sgRNA 1	GAACACGCTCGACGACGTGT CGG	IDT
sgRNA 2	GAATGGCGCTACATTTCCCT TGG	IDT

**Table:** 

PCR Primer	Sequence (5’→3’)
Daphnia *scarlet* 5	CTACTGATGGATCACCTCCACTCGTTA
Daphnia *scarlet* 3	CATTAAAGCCACGAGCGAACCGGGTTG
